# Cnidarians are CLOCKing in

**DOI:** 10.7554/eLife.98512

**Published:** 2024-05-08

**Authors:** Erica R Kwiatkowski, Patrick Emery

**Affiliations:** 1 MD/PhD graduate program, University of Massachusetts Chan Medical School Worcester United States; 2 Department of Neurobiology, University of Massachusetts Chan Medical School Worcester United States

**Keywords:** *Nematostella vectensis*, circadian rhythms, genetic mutant, behavior, transcriptome, light-pathway, Other

## Abstract

Studies of the starlet sea anemone provide important insights into the early evolution of the circadian clock in animals.

**Related research article** Aguillon R, Rinsky M, Simon-Blecher N, Doniger T, Appelbaum L, Levy O. 2023. CLOCK evolved in cnidaria to synchronize internal rhythms with diel environmental cues. *eLife* **12**:RP89499. doi: 10.7554/eLife.89499.

The concept of circadian rhythms is quite intuitive: life on Earth needs to be able to anticipate predictable changes in the environment – and take advantage of them. Thus, in most species, a wide range of bodily functions and behaviors are optimized to the time-of-day. Moreover, the disruption of circadian rhythms diminishes survival in the wild, and increases the risk of many diseases in humans (Lane et al., 2023). The molecular clocks that generate circadian rhythms have, therefore, been the focus of much research and their mechanisms are now well understood.

In animals, circadian clocks have predominantly been studied in fruit flies (*Drosophila melanogaster*) and mice. The mechanisms involved in these two species are remarkably similar (Weaver and Emery, 2013). Briefly, a dimer comprised of CLOCK (CLK) and CYCLE (CYC, called BMAL1 in mammals) acts as a transcription factor and promotes the expression of its own repressors: in mammals these repressors are the PERIOD (PER) and Cryptochrome (CRY) proteins, and in *Drosophila* they are PER and TIMELESS (Figure 1A). This negative feedback loop keeps oscillating with a period of ~24 hours, even under constant conditions, and drives the rhythmic expression of many genes (Li and Zhang, 2015).

**Figure 1.**
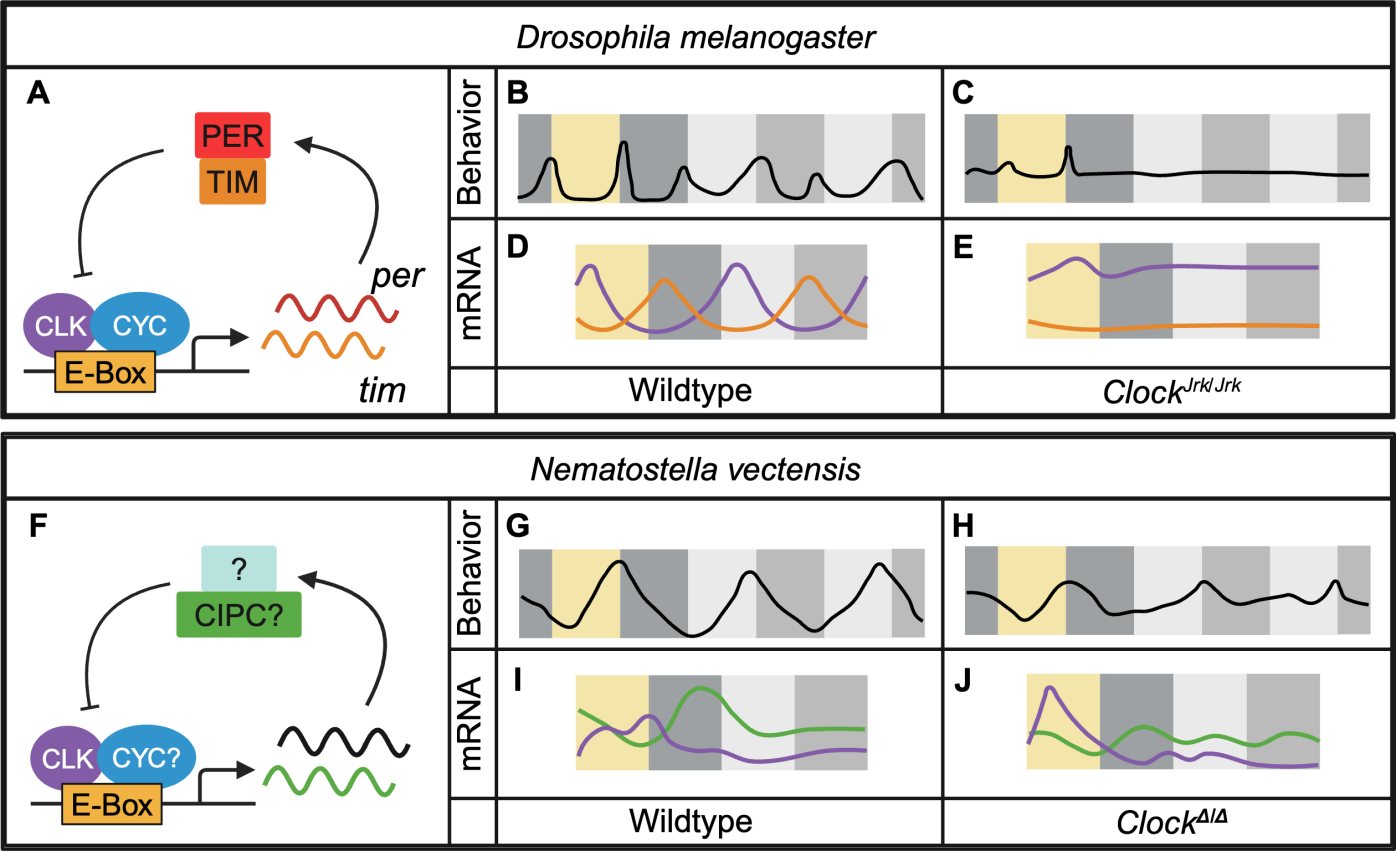
Comparison of circadian rhythms in *Drosophila melanogaster* and *Nematostella vectensis*. (**A**) In *Drosophila melanogaster*, the proteins CLOCK (CLK) and CYCLE (CYC) bind to E-boxes to promote expression of the genes that code for PERIOD (PER) and TIMELESS (TIM), which go on to repress CLK/CYC activity. (**B**) Locomotor behavior of flies under cycles of light and darkness (yellow and dark grey shadings) is strongly rhythmic, with increased activity in anticipation of dawn and dusk. Under conditions of constant darkness (light grey and dark grey shadings), activity is still rhythmic, with a similar bimodal pattern. (**C**) Flies homozygous for a mutant version of the gene that codes for CLK (*Clock^Jrk^*) acutely respond to the light and dark transitions, but become immediately arrhythmic under constant darkness. (**D**) The mRNA transcripts for circadian clock genes, including CLK (purple) and PER (orange), are rhythmic both under cycles of light and darkness, and under constant darkness. (**E**) However, these rhythms are lost in mutant flies under both sets of conditions. (**F**) In the anemone *Nematostella vectensis*, CLK (and possibly CYC) binds to E-boxes to promote the rhythmic expression of the gene that codes for a protein called CIPC (green), and possibly other circadian genes (black). The results of Aguillon et al. suggest that CIPC might repress CLK-driven transcription to form a feedback loop. (**G**) Under cycles of light and darkness, wild-type anemones exhibit rhythmic activity which persists under constant darkness. (**H**) Anemones homozygous for a mutant version of the *Clock* gene (*Clock^Δ^*) still respond to cycles of light and darkness, but this rhythmicity is lost upon transition to constant darkness. (**I**) Under cycles of light and darkness, the mRNA transcripts for CLK (violet) and its proposed repressor CIPC (green) oscillate in wild-type anemones. These oscillations are out of phase with each other, and are immediately lost upon transition to constant darkness. (**J**) Anemones homozygous for a mutant version of the *Clock* gene show abnormally phased, light-dependent rhythms of the mRNA transcripts for CLK, whereas the rhythms of the mRNA transcripts for CIPC are severely compromised.

Image credit: Created with BioRender.com.

*Drosophila* and mice both belong to the clade Bilateria, but they are separated by 600 million years of evolution. The similarity between their circadian clock mechanisms raises the following fundamental question: when did the animal circadian clock emerge during evolution? Was it before or after the divergence of bilaterians and a related phylum known as the cnidarians? Now, in eLife, Raphael Aguillon, Mieka Rinsky, Oren Levy and colleagues at Bar-Ilan University report the results of experiments on *Nematostella vectensis* – a cnidarian known as the starlet sea anemone – that shed light on this critical question (Aguillon et al., 2023).

Circadian rhythms of both behavior and gene expression have previously been observed in experiments on this species, which carries homologs of the CLK, CYC and CRY proteins but not, intriguingly, of PER proteins (Reitzel et al., 2010; Hendricks et al., 2012). Thus, to determine whether the mechanism that underpins the circadian clock in *N. vectensis* is related to that found in bilaterians, Aguillon et al. generated a mutant allele of the gene that codes for CLK. Under cycles of light and darkness they found that both wild-type anemones and mutant anemones were nocturnal (Figure 1G and H). Next they recorded the activity of the anemones under conditions of constant darkness: as expected, wild-type anemones exhibited prominent rhythms of activity with a period of ~22 hours. On the other hand, virtually all the mutant animals were arrhythmic. Thus, the role of CLK in controlling circadian behavior is conserved between cnidarians and bilaterians (Figure 1B, C, G and H).

Aguillon et al. then tested how the loss of CLK activity impacted rhythmic gene expression in *N. vectensis*, with intriguing results. First, consistent with the arrhythmic behavior of mutant animals, the vast majority of genes that were rhythmic in wild-type animals, whether under light-dark cycles or constant darkness, were no longer rhythmic in mutant animals (Figure 1I and J). Surprisingly, however, there were still rhythmic genes in the mutants, even under constant darkness. Thus, there is a mechanism that generates distinct 24 hour rhythms of gene expression that is independent of CLK. Also, and rather curiously, there was almost no overlap between the transcripts that were rhythmic under light-dark cycles and those that were rhythmic under constant darkness, regardless of genotype (Figure 1I and J). This suggests that exposure to light might completely override the circadian control of gene expression. Alternatively, light and the circadian clock(s) might impact distinct tissues, with light-dependent rhythms obscuring circadian ones.

In summary, Aguillon et al. provide evidence for a very early evolutionary recruitment of CLK in the circadian clock. However, since the cnidarian clock does not rely on PER, we do not know the identity of the repressive molecules involved. CRY2 is an obvious candidate because it resembles the CRY proteins found in the mammalian circadian clock (Reitzel et al., 2010). Aguillon et al. also point to the presence of CIPC (Figure 1F) – a protein that is known to modulate circadian rhythms through the repression of CLK/CYC in mammals and *Drosophila* (Rivas et al., 2021; Zhao et al., 2007).

Intriguingly, under cycles of light and darkness, the mRNA transcripts for CLK and CIPC both oscillate, but these oscillations are out of phase with each other (Figure 1I), reminiscent of the antiphase mRNA oscillations of activators and repressors in flies and mammals (Figure 1D; Weaver and Emery, 2013). However, these two transcripts (as well as the transcripts for other circadian clock gene candidates) become arrhythmic under conditions of constant darkness. Perhaps only a subset of tissues, like neurons driving rhythmic behavior, have a robust, self-sustained circadian oscillator. If so, the oscillations in the mRNA transcripts for CLK and CIPC might not be detectable when mRNA levels are measured in the whole organism, as Aguillon et al. did.

This would not be so different from *Drosophila*: the oscillations in many peripheral tissues quickly decrease in amplitude upon transition to constant darkness, but in circadian neurons they remain robustly rhythmic in order to drive rhythmic behavior (Johnstone et al., 2022; Stanewsky et al., 1997). Alternatively, the *N. vectensis* clock might rely heavily on post-transcriptional regulation. This brings to mind the cyanobacteria *Synechoccus elongatus*, in which transcriptional rhythms are dispensable for circadian oscillations, while a phosphorylation cycle with a period of 24 hours can be observed even in vitro (Nakajima et al., 2005).

Considerable work will be needed to elucidate the mechanisms of circadian rhythms in anemones and other cnidarians. First, the remaining core clock genes need to be identified. A CLK-independent oscillator appears to be present, but its nature is completely unknown. The relationship between light-driven rhythms and CLK-driven rhythms will also need to be sorted out (Figure 1I and J), especially the question of which tissues are impacted by these rhythms. Aguillon et al. have elegantly demonstrated that *Nematostella vectensis* is a potent model to answer these fascinating questions and shed light on the origin of circadian clocks in animals.
